# Blood Pressure Control Among Non-Hispanic Black Adults Is Lower Than Non-Hispanic White Adults Despite Similar Treatment With Antihypertensive Medication: NHANES 2013–2018

**DOI:** 10.1093/ajh/hpac011

**Published:** 2022-06-16

**Authors:** Donald K. Hayes, Sandra L. Jackson, Yanfeng Li, Gregory Wozniak, Stavros Tsipas, Yuling Hong, Angela M. Thompson-Paul, Hilary K. Wall, Cathleen Gillespie, Brent M. Egan, Matthew D. Ritchey, Fleetwood Loustalot

**Affiliations:** 1 Division for Heart Disease and Stroke Prevention, Centers for Disease Control and Prevention, Atlanta, Georgia, USA;; 2 Improving Health Outcomes, American Medical Association, Chicago, Illinois, USA;; 3 National Heart, Lung, and Blood Institute, National Institutes of Health, Bethesda, Maryland, USA.

**Keywords:** antihypertensive medication, blood pressure, control, disparities, hypertension, NHANES

## Abstract

**BACKGROUND:**

Controlled blood pressure can prevent or reduce adverse health outcomes. Social and structural determinants may contribute to the disparity that despite equivalent proportions on antihypertensive medication, non-Hispanic Black (Black) adults have lower blood pressure control and more cardiovascular events than non-Hispanic White (White) adults.

**METHODS:**

Data from 2013 to 2018 National Health and Nutrition Examination Survey were pooled to assess control among Black and White adults by antihypertensive medication use and selected characteristics using the 2017 American College of Cardiology/American Heart Association (ACC/AHA) Blood Pressure Guideline definition (systolic blood pressure <130 mm Hg and diastolic blood pressure <80 mm Hg) among 4,739 adults.

**RESULTS:**

Among those treated with antihypertensive medication, an estimated 34.9% of Black and 45.0% of White adults had controlled blood pressure. Control was lower for Black and White adults among most subgroups of age, sex, education, insurance status, usual source of care, and poverty–income ratio. Black adults had higher use of diuretics (28.5%—Black adults vs. 23.5%—White adults) and calcium channel blockers (24.2%—Black adults vs. 14.7%—White adults) compared with White adults. Control among Black adults was lower than White adults across all medication classes including diuretics (36.1%—Black adults vs. 47.3%—White adults), calcium channel blockers (30.2%—Black adults vs. 40.1%—White adults), and number of medication classes used.

**CONCLUSIONS:**

Suboptimal blood pressure control rates and disparities warrant increased efforts to improve control, which could include addressing social and structural determinants along with emphasizing implementation of the 2017 ACC/AHA Blood Pressure Guideline into clinical practice.

## GRAPHICAL ABSTRACT

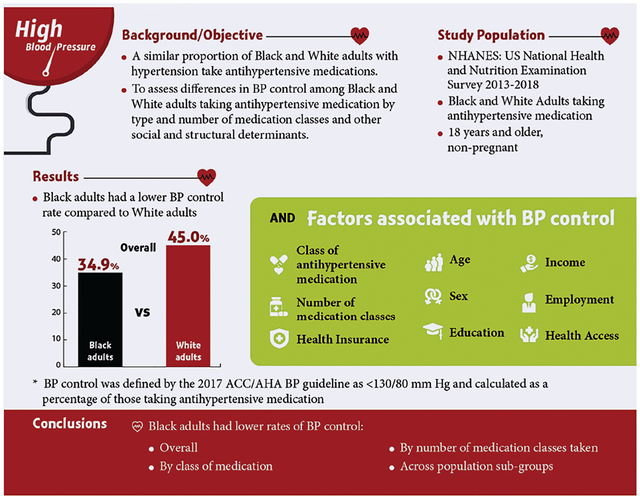

Hypertension is an important risk factor for cardiovascular disease, the leading cause of death in the United States and globally.^1^ Appropriate control of hypertension can reduce heart attacks, heart failure, strokes, chronic kidney disease, and all-cause mortality.^2–4^ During 2001–2010, partly attributed to an increased emphasis on combination therapy (i.e., prescribing more than 1 medication class) and single-pill combinations (i.e., using more than 1 medication combined into a single pill to simplify dosing regimen), overall blood pressure control levels significantly improved.^5,6^ However, there has been little to no improvement in blood pressure control trends from 2010 through 2016 among most populations.^7^ During 2017–2018, there were declines in blood pressure control compared with prior years with differences among population subgroups.^8^ Among non-Hispanic Black (Black) adults, who have nearly equivalent treatment levels (i.e., measured by being on antihypertensive medication) to non-Hispanic White (White) adults, control rates are consistently much lower.^7,9^ To reduce disparities in the prevalence of blood pressure control, and its sequelae, it is important to assess factors that may be associated with lower control rates to inform interventions to improve outcomes.

Although race-specific prescribing patterns have been observed at least since 2003,^10^ it was not until 2014 when an expert panel recommended specific antihypertensive medication regimens to improve control rates among Black adults.^11^ For example, diuretics and calcium channel blockers were promoted as first-line therapy among Black adults and multiple medication classes are often started at the same time. Despite these recommendations, there has been limited improvement in the disparity in blood pressure control between Black and White adults.^8,10^ Hypertension management recommendations were further updated in the 2017 American College of Cardiology/American Heart Association Guideline for the Prevention, Detection, Evaluation, and Management of High Blood Pressure in Adults (2017 ACC/AHA Blood Pressure Guideline), which defined controlled hypertension as systolic blood pressure <130 mm Hg and diastolic blood pressure <80 mm Hg.^12^ In this guideline, the specific prescribing recommendations for Black adults are continued from the expert panel recommendations in 2014. There are limited data on race disparities in blood pressure control based on the thresholds used in the 2017 ACC/AHA Blood Pressure Guideline.

This analysis uses pooled data from 3 cycles (2013–2018) of the National Health and Nutrition Examination Survey (NHANES) to examine differences in blood pressure control using the 2017 ACC/AHA Blood Pressure Guideline among the Black and White adult population with hypertension by selected characteristics, antihypertensive medication class, and number of medication classes.

## METHODS

NHANES is a biennial survey conducted among civilian, noninstitutionalized residents of the United States. Additional details about the design and content of NHANES are available online (http://www.cdc.gov/nchs/nhanes.htm). This analysis combined 3 survey cycles 2013–2018 to allow sufficient sample size to generate stable estimates. We limited the analysis to the 9,856 nonpregnant adults aged >18 years who self-identified as White or Black and did not report a Hispanic ethnicity. We excluded those who did not have a measured blood pressure (*n* = 376), were missing prescription medication information (e.g., self-reported antihypertensive medication but none included in medication file; or were identified as using antihypertensive medication based on the medication file, but did not self-report use of antihypertensive medication; or did not answer question on self-reported use of antihypertensive medications; *n* = 731), and those who were missing information on healthcare access, health insurance coverage, education level, or employment status (*n* = 72) to obtain a final analytic sample of 8,677 adults. Of these, 4,739 adults were determined to have hypertension and included in this analysis.

Blood pressure measurements were obtained with mercury sphygmomanometers following specific protocols.^13^ All available measurements (0.2% had 1, 0.9% had 2, 97.8% had 3, and 1.2% had 3 systolic readings with only 1–2 diastolic readings) were used to determine average blood pressure. Based on the 2017 ACC/AHA Blood Pressure Guideline, a respondent was identified as having hypertension based on an average systolic blood pressure ≥130 mm Hg, a diastolic blood pressure ≥80 mm Hg, or current use of antihypertensive medication based on both self-report and review of medications provided during the interview component. Blood pressure control among those on antihypertensive medication was defined by systolic blood pressure <130 mm Hg and diastolic blood pressure <80 mm Hg.^12^ These definitions were applied to all cycles of data.

Antihypertensive agents identified through review of the medication file were categorized into the following classes: (i) Diuretics as a group which included thiazide, loop, and potassium-sparing, (ii) beta-blockers (BBs), (iii) calcium channel blockers (CCBs), (iv) angiotensin-converting enzyme inhibitors or angiotensin II receptor blockers (ACEIs or ARBs), and (v) other antihypertensive agents (including alpha 1-blockers, central alpha 2-agonists, direct vasodilators, renin inhibitors, and other centrally acting drugs). The prevalence of use by medication class among those on any antihypertensive regimen and by number of medication classes used (1, 2, 3, 4, or 5 classes) was calculated. The number of antihypertensive medication classes, combinations of classes, and respective control status were reported.

Estimates of blood pressure control for Black and White adults were calculated by age groups (18–44, 45–64, and >65 years), sex, and other characteristics often referred to as proxy social determinant of health measures including healthcare access (e.g., usual source of care, number of doctor visits in past year), health insurance coverage (e.g., public, private, and none), employment status (e.g., employed for wages vs. all others), educational level based on self-reported highest grade or degree completed (e.g., <high school, high school—includes graduate equivalent degree, some college—includes associate degree, and college graduate), and poverty–income ratio (PIR). The PIR is based on a comparison of family income with the poverty threshold determined by the US Census Bureau. The PIR values were stratified into 3 commonly used categories: PIR <1.0 (low income), 1.0 ≤ PIR ≤ 3.0 (middle income), and ≥3.0 (high income) and those with missing, refused, or unknown PIR were maintained as a category (1.6% among Black adults and 4.5% among White adults).

Statistical analyses were conducted using SAS (version 9.4) and SUDAAN (version 11) software to account for sampling weights and to adjust variance estimates for the multistage, clustered sample design. Chi-square tests were used to determine differences by population characteristics among those with hypertension. Consistent with National Center for Health Statistics (NCHS) recommendations, population estimates for the individual characteristics were based on the prevalence of the characteristics and the average of number of Black or White adults reflected in the 2013–2018 American Community Survey data file. To assess differences between race groups for blood pressure control overall and within the selected characteristics accounting for age and sex differences, separate multivariate logistic regression analyses with blood pressure control as the dependent variable and race and the selected characteristic as the independent variables were performed with adjustment for age (3 level categories: 18–44, 45–64, and 65 and older) and sex. Similarly, separate multivariate logistic regression analyses were performed with class and number of antihypertensive medications used as the dependent variable, race and the selected characteristics as the independent variables with adjustment for age and sex were used to detect race group differences by the selected characteristics. As the time frame of the data overlapped the guideline implementation and to allow a degree of comparison between the old and the new 2017 ACC/AHA Blood Pressure Guideline, separate analyses based on the older threshold of <140/90 mm Hg are included in Supplementary Tables online. This supplemental analysis is a subset of the full analyses due to the higher threshold of 140/90 mm Hg used to define an individual with hypertension and to assess blood pressure control. Additionally, the same method was used to define someone on antihypertensive medication based on both self-report and from the medication provided during the interview component as was done for the primary analysis. Estimates were suppressed when not stable based on NCHS data suppression guidelines for proportions.^14^ A *P* value of <0.05 defined statistical significance. All NHANES participants provided informed consent, and the survey received ethical approval through the Research Ethics Review Board of NCHS.

## RESULTS

**Figure 1.**
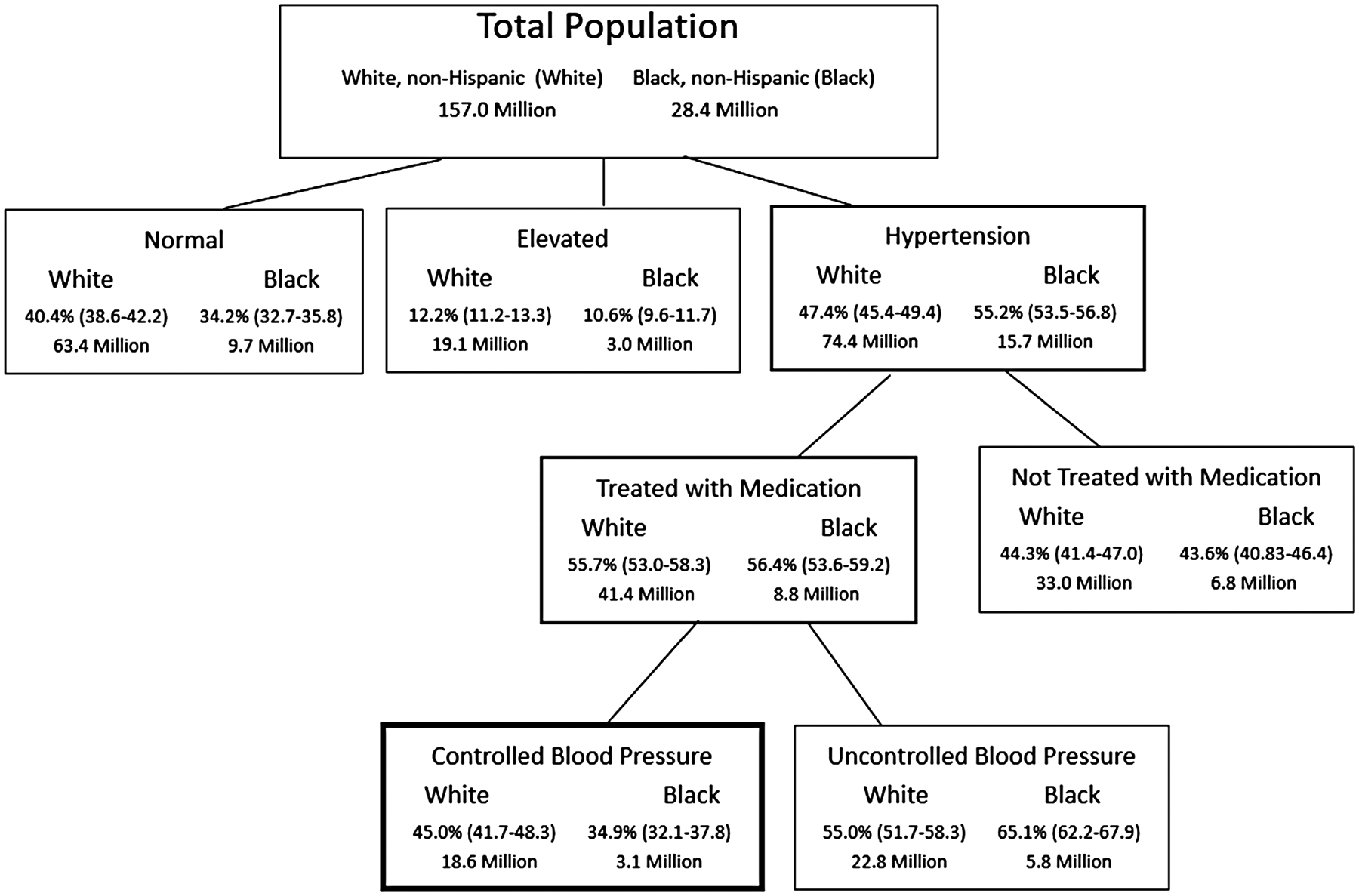
Hypertension cascade among non-Hispanic White and non-Hispanic Black adults aged ≥18 years—National Health and Nutrition Examination Survey (NHANES) 2013–2018. Notes: Includes White and Black adults (≥18 years) not selecting Hispanic as an ethnicity. Normal blood pressure is defined as an average systolic blood pressure <120 mm Hg and an average diastolic blood pressure <80 mm Hg. Elevated blood pressure is defined as an average systolic blood pressure 120–129 mm Hg and an average diastolic blood pressure <80 mm Hg. Hypertension is defined as an average systolic blood pressure ≥130 mm Hg or an average diastolic blood pressure ≥80 mm Hg or self-reported current use of blood pressure lowering medication and identified with antihypertensive medication from medication file.

During 2013–2018, an estimated 55.2% of Black adults (15.7 million individuals) and 47.4% of White adults (74.4 million) had hypertension (Figure 1). Among adults with hypertension, 56.4% of Black adults (8.8 million) and 55.7% of White adults (41.4 million) were on antihypertensive medication, yet among those only 34.9% of Black adults (3.1 million) and 45.0% of White adults (18.6 million) had blood pressure controlled.

Black and White adults with hypertension differed across nearly all characteristics. Black adults were more likely to be younger (aged 18–64 years) than White adults (78.2% for Black vs. 63.9% for White; and 30.4% vs. 19.6% for subgroup aged 18–44 years) and more likely to be female (54.0% vs. 47.4%; Table 1). Black adults were less likely to have health insurance (82.6% vs. 91.9% for any health insurance, and 46.0% vs. 69.2% for private health insurance), a usual source of care (86.8% vs. 90.3%), have less than a high school education (19.0% vs. 9.0%), and a PIR ≥3.0 (27.9% vs. 53.5%).

The prevalence of blood pressure control was lower in Black adults compared with White adults for nearly all characteristics evaluated. Among those aged 18–64 years, Black adults had lower estimates of control compared with White adults (36.0% vs. 49.2%) as well as among the subgroups aged 45–64 years (35.6% vs. 49.2%) and 18–44 years (37.6% vs. 48.9%; Table 2). Estimates of control were also lower among Black than White adults who were 65 years and older (32.4% vs. 40.8%), male (33.5% vs. 44.6%), and female (35.8% vs. 45.3%). For healthcare access and utilization, blood pressure control was also lower among Black than White adults, including those with health insurance (35.2% vs. 45.1% for any insurance; 36.1% vs. 46.3% for private insurance), having a usual source of healthcare (35.1% vs. 45.3%), and by number of doctor visits (36.3% vs. 46.7% for 2–3 visits; 36.5% vs. 45.6% for 4 or more visits). Other social determinants of health indicators with lower blood pressure control estimates among Black adults compared with White adults included having a 1.0–3.0 PIR (29.9% vs. 39.0%), a PIR >3.0 (38.2% vs. 50.7%), having some college education (32.3% vs. 48.1%), college graduate (38.7% vs.42.6%), and not being employed (34.9% vs. 43.7%).

The prevalence of using any antihypertensive medication overall (56.4% vs. 55.7%) was lower among Black compared with White adults, CCB (24.2% vs. 14.7%), and diuretic (28.5% vs. 23.5%) use was higher among Black and White adults (Table 3). The 2 most common medication classes used by both Black and White adults were ACEI or ARB (36.8% for Black adults vs. 41.4% for White adults) and diuretics (28.5% vs. 23.5%). Among those using just 1 medication class, Black adults had higher use of CCB (4.8% vs. 1.9%) and lower use of ACEI or ARB (8.4% vs. 13.3%) and BB (2.4% vs. 4.1%) than White adults. Among those using 2 medication classes, Black adults had higher estimates of using 2 medication classes (21.1% vs. 20.4%) and higher estimates of combinations of CCB and diuretics (2.2% vs. 0.8%) and CCB and BB (2.0% vs. 0.9%) compared with White adults. Among those on 3 medication classes, Black adults had higher estimates of using 3 medication classes (10.5% vs. 9.4%) and higher estimates of using the combination of ACEI or ARB/CCB/diuretic (3.9% vs. 1.7%) compared with White adults. Among those using 4 or more medication classes, Black adults had higher estimates of using 4 or more medication classes (5.7% vs. 4.3%) and higher estimates of using the combination of ACEI or ARB/BB/CCB/Diuretic/Other (0.9% vs. 0.3%) compared with White adults.

**Figure 2.**
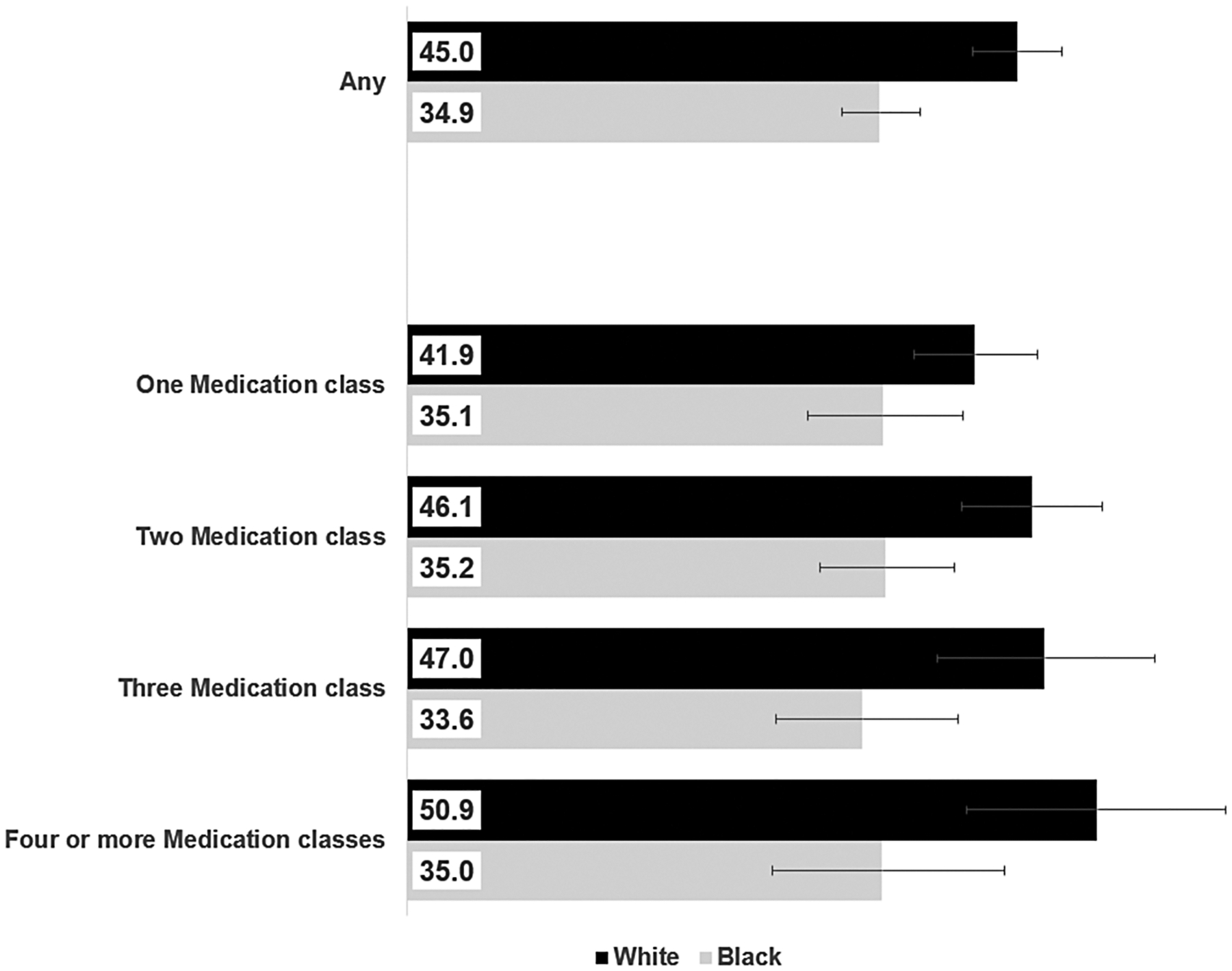
Prevalence of blood pressure control (<130/80 mm Hg) by use of any antihypertensive medication and by number of medication classes among White and Black adults—National Health and Nutrition Examination Survey (NHANES) 2013–2018. Note: 95% confidence intervals around prevalence estimate are reflected as error bars. White and Black adults are limited to those not selecting Hispanic as an ethnicity.

Among those taking any antihypertensive medication, Black adults had a lower prevalence of blood pressure control compared with White adults for every class, ranging from 30.2% to 36.5% for Black adults and 40.1% to 47.3% for White adults (Table 3). The prevalence of blood pressure control among Black adults with number of medication classes ranged from 33.6% in those on 3 classes to 35.2% in those on 2 classes, whereas control among White adults taking more medication classes increased from 41.9% in those on 1 medication class to 50.9% in those on 4 or more classes (Figure 2). Black adults also had lower control estimates compared with White adults by number of medication classes.

Based on the older threshold of 140/90 mm Hg, Black adults had a lower prevalence of blood pressure control than White adults (58.6% vs. 69.7%; Supplementary Table S1 online). Additionally, Black adults had lower prevalence of blood pressure control than White adults for all characteristic subgroups demonstrated above based on the 130/80 mm Hg threshold. Additionally, Black adults who had less than a high school education (53.3% vs. 71.0%) and those with a high school education (58.2% vs. 67.9%), and those with a PIR <1.0 (56.4% vs. 70.8%) had lower estimates of blood pressure control than White adults. Among those on each class of medication and by number of medication classes, Black adults had lower estimates of blood pressure control compared with White adults (Supplementary Table S2 online).

## DISCUSSION

This analysis highlighted overall low blood pressure control rates using the 2017 ACA/AHA Blood Pressure Guideline and demonstrated racial disparities with consistently lower levels among Black adults when compared with White adults overall, by medication class, by number of medication classes used, and among most population subgroups including age, sex, healthcare access, education, and family poverty. Despite greater use of diuretics and CCB reported among Black adults consistent with the 2017 ACA/AHA Blood Pressure Guideline and first recommended in 2014, blood pressure control rates remained lower with these and all medication classes when compared with White adults. Almost the exact same findings but of different magnitude were found when the older guidelines using less stringent blood pressure thresholds were applied. To improve blood pressure control and reduce its disparities, it is important to understand and address potential barriers, particularly social and structural determinants influencing blood pressure control across all groups.

Use of antihypertensive medication is an important contributor to blood pressure control that depends on access to healthcare as well as other factors that influence blood pressure and use of antihypertensive medications. Clinical strategies that promote blood pressure control typically include frequent healthcare interaction and medication management such as intensification of antihypertensive medication to reach control.^15^ Social and structural determinants of health may impact the use of antihypertensive medication through medication adherence, or taking medication as prescribed. Medication adherence depends on factors such as access to and affordability of medication, associated visit costs, health literacy, and appropriate community partnerships to ensure equitable access.^12^ Poverty, healthcare and medication costs, and health literacy also affect medication adherence and ability to access clinical care for blood pressure, including in-person healthcare visits.^16^ Blood pressure tracking outside of the clinical setting such as those promoted in self-measured blood pressure monitoring programs may improve control as they increase awareness and potential for management of the condition beyond the clinical setting.^17–19^ A recent study assessing some social determinants demonstrated that Black adults, not having a regular source of healthcare, being unemployed, not having insurance were associated with lower blood pressure control.^20^ Additionally, not having a doctor visit in the past year has been associated with lower blood pressure control.^21^ In the current study, Black adults consistently had lower rates of control across nearly every subpopulation even among those taking the guideline recommended medication classes consistent with much of the published literature. Another paper using NHANES data from 2015 to 2018 demonstrated a 20% lower odds of having controlled blood pressure among Black adults compared with White adults along with differences by age and health insurance, but those related to education, sex, income, and health visit in past year were no longer significant in the fully adjusted model.^8^ Racial differences are complex and require exploration of interconnected factors such as social and structural determinants.

Black adults tend to develop hypertension earlier and more severely than White adults, with disparities already seen in those <30 years.^22^ Black adults are also known to have more difficult to treat hypertension, sometimes called treatment resistant hypertension, and often require 3 or more medications for control.^23–25^ It is unclear if these physiological differences are due to genetic differences or to underlying social and structural determinants.^26–28^ Additionally, determinants related to inability to access healthcare and appropriate treatment for Black adults could also contribute to decreased control. For example, structural and social determinants may be related to factors such as the interaction between a patient and their provider as shown in a recent study where a strong relationship was shown to improve medication adherence and blood pressure control among Black adults.^29^ Our analysis found an increased use of 4 or more medication classes among Black adults but no difference in blood pressure control when compared with Black adults on fewer medications. This agrees with apparent treatment resistant hypertension but understanding if this is related to structural or social determinants is not clear. The increased prevalence at younger ages for Black adults, highlights the importance of identification and appropriate control with antihypertensive medication and lifestyle modifications as early in the life course as possible.^30,31^ Additionally, several interventions demonstrating improved blood pressure control among Black adults have centered on increasing community engagement of patients in settings such as barbershops and faith-based institutions as well as use of technology such as self-measured blood pressure monitoring.^32–35^ Thus, diverse strategies are needed to improve blood pressure control, including lifestyle changes and optimizing medication use per the 2017 ACC/AHA Blood Pressure Guideline and developing a deeper understanding of factors that may influence the use of medication and lifestyle changes. The World Health Organization has defined social determinants of health as the “circumstances in which people are born, grow, live, work, and age and the systems put in place to deal with illness.”^36^ The impact of social determinants on blood pressure control is not well characterized though an AHA Scientific Statement discussed the importance of considering their impact on risk factors and outcomes for cardiovascular disease.^37^ Ways to reduce disparities are highlighted in the Surgeon General’s Call to Action to Control Hypertension in 2020 emphasized the importance of addressing hypertension through a broad framework to address social determinants including activating and equipping business, education, and faith-based institutions as a critical step to improve blood pressure control.^38^

This study has at least 5 limitations. As the data collected are cross-sectional in nature, information on the longitudinal course of disease including duration with hypertension is unknown. Secondly, there are limited measures of antihypertensive medication history and patterns of use. Other data sources such as clinical records and linked claims data may allow a more definitive evaluation of blood pressure control not available in NHANES including longitudinal prescribing patterns, medication dosage, intensification of medication, and medication adherence. Thirdly, this analysis applied the 2017 ACC/AHA Blood Pressure Guideline on older data when clinical judgment/practice was based on less stringent control levels. Thus, this analysis likely underestimates blood pressure control prevalence particularly for the earlier period (2013–2017) when older guidelines were used. However, calculations using the prior definition of hypertension and blood pressure control (<140/90 mm Hg) demonstrated similar disparities among the same population subgroups and some additional ones with Black adults having lower estimates compared with White adults. However, the analysis using the older threshold to define hypertension and the assessment of control in this study didn’t include comorbidities which could impact the observed control rates due to lower thresholds for those with specific comorbidities. Fourth, this analysis focused just on blood pressure control in Black compared with White adults so Asian, Hispanic, and those that report more than 1 race were not included in the analysis. Lastly, this analysis compared estimates between Black and White adults for selected characteristics with adjustment for age and sex differences, thus other factors that could be accounted for in a full multivariate model were not included in testing for differences.

Overall, about half of Black and White adults have hypertension, and nearly 3 out of 5 are treated with antihypertensive medication. Blood pressure control rates are suboptimal with approximately one-third of Black adults and two-fifths of White adults currently taking antihypertensive medication having blood pressure controlled to <130/80 mm Hg. As a result, among those treated for hypertension, an estimated 22.8 million White adults and 5.7 million Black adults have uncontrolled blood pressure. These overall suboptimal blood pressure control rates and the persistent disparities for Black adults as compared with White adults across nearly all subgroups included in this analysis warrant increased efforts to improve control of blood pressure. These could include more widespread adoption by clinicians and their patients of the current 2017 ACC/AHA Blood Pressure Guideline and responding to the Surgeon General’s Call to Action through the development and implementation of multisectoral partnerships that address important social and structural determinants of blood pressure control.

## Supplementary Material

SupplementaryOnlineTables

## Figures and Tables

**Table 1.** Characteristics of White and Black adults with hypertension—National Health and Nutrition Examination Survey (NHANES) 2013–2018

	White (*n* = 2,754, *N* = 74.4 million)			Black (*n* = 1,985, *N=* 15.7 million)			
	*N* (millions)	%	(95% CI)	*N* (millions)	%	(95% CI)	*P* value
Total	74.4	100.0		15.7	100.0		
Age group (years)							<0.01
18–64	47.6	63.9	(61.7–66.1)	12.2	78.2	(75.8–80.4)	
18–44	14.6	19.6	(17.8–21.6)	4.8	30.4	(27.3–33.6)	
45–64	33.0	44.3	(42.4–46.2)	7.5	47.8	(44.4–51.3)	
65+	26.9	36.1	(33.9–38.3)	3.4	21.8	(19.6–24.2)	
Sex							<0.01
Men	39.1	52.6	(50.4–54.8)	7.2	46.0	(44.2–47.8)	
Women	35.3	47.4	(45.2–49.6)	8.5	54.0	(52.2–55.8)	
Insurance							<0.01
Yes	68.4	91.9	(89.6–93.7)	12.9	82.6	(80.1–84.9)	
Public	16.9	22.7	(20.5–25.1)	5.7	36.6	(33.3–40.1)	
Private	51.5	69.2	(66.2–72.1)	7.2	46.0	(42.7–49.3)	
No	6.0	8.1	(6.3–10.4)	2.7	17.4	(15.1–19.9)	
Usual source for care							0.02
Yes	67.2	90.3	(88.7–91.7)	13.6	86.8	(84.1–89.1)	
No	7.2	9.7	(8.3–11.3)	2.1	13.2	(10.9–15.9)	
Number of doctor visits in past year							0.02
0 visits	7.1	9.5	(8.2–11.1)	2.0	12.8	(11.3–14.4)	
1 visit	9.9	13.3	(11.4–15.6)	2.4	15.5	(13.8–17.3)	
2–3 visits	23.2	31.2	(29.0–33.5)	4.7	30.1	(27.7–32.6)	
4+ visits	34.2	45.9	(43.1–48.8)	6.5	41.6	(38.9–44.4)	
Education level							
<High school	6.7	9.0	(7.7–10.5)	3.0	19.0	(16.4–21.9)	<0.01
High school	21.8	29.4	(25.9–33.0)	3.0	19.0	(16.5–21.7)	
Some college	26.6	35.8	(33.3–38.3)	5.2	33.1	(30.6–35.7)	
College graduate	19.2	25.8	(23.5–28.3)	4.5	28.9	(26.8–31.1)	
Poverty–income ratio							<0.01
<1.0	5.5	7.4	(5.9–9.2)	3.5	22.6	(19.9–25.6)	
1.0–3.0	24.4	32.8	(30.1–35.6)	6.0	38.3	(34.8–42.0)	
>3.0	39.8	53.5	(49.6–57.4)	4.4	27.9	(24.0–32.2)	
Missing	4.7	6.3	(5.0–7.9)	1.7	11.1	(8.9–13.8)	
Employment status							0.21
Yes	38.1	51.1	(48.1–54.2)	8.4	53.4	(51.0–55.8)	
No	36.4	48.9	(45.8–51.9)	7.3	46.6	(44.2–49.0)	

Notes:

(1) Hypertension status based on self-reported use of antihypertensive medication or blood pressure ≥130/80 mm Hg.

(2) Includes nonpregnant adults age ≥18 years with complete data on hypertension status.

(3) *n* = unweighted population size used in analysis; 95% CI = 95% confidence interval around estimate.

(4) *N*: annual population in millions, calculated from the American Community Survey data released by NCHS, averaged across the 3 cycles. For additional information, see https://wwwn.cdc.gov/nchs/nhanes/ResponseRates.aspx#population-totals.

(5) Poverty–income ratio (PIR) is based on comparison of family income with the poverty threshold determined by the US Bureau of Census. The PIR values were stratified into categories: PIR < 100% (low income), 1,300% ≤ PIR ≤ 300% (middle income), and ≥300% (high income) and those with missing, refused, or unknown were maintained as a category (missing).

(6) Employment status value of “Yes” refers to Employed for wages and value of “No” refers to all others.

(7) *P* value of chi-square test for the association between characteristics and race–ethnicity.

(8) White and Black adults are limited to those not selecting Hispanic as an ethnicity.

**Table 2.** Prevalence of blood pressure control (<130/80 mm Hg) among White and Black adults with hypertension currently taking medication—National Health and Nutrition Examination Survey (NHANES) 2013–2018

	White (*N* = 41.4 million)			Black (*N* = 8.8 million)			
	*N*	%	(95% CI)	*N*	%	(95% CI)	*P* value
Total	18.6	45.0	(41.7–48.3)	3.1	34.9	(32.1–37.8)	<0.01
Age group (years)							
18–64	10.2	49.2	(43.8–54.6)	2.1	36.0	(32.7–39.6)	<0.01
18–44	1.6	48.9	(39.5–58.4)	0.5	37.6	(31.2–44.5)	<0.01
45–64	8.5	49.2	(43.0–55.4)	1.7	35.6	(31.7–39.7)	<0.01
65+	8.5	40.8	(36.5–45.3)	0.9	32.4	(28.5–36.5)	<0.01
Sex							
Men	7.6	44.6	(40.0–49.3)	1.0	33.5	(30.0–37.1)	<0.01
Women	8.6	45.3	(41.3–49.4)	1.6	35.8	(31.7–40.1)	<0.01
Health insurance							
Yes	17.9	45.1	(41.9–48.3)	2.8	35.2	(32.3–38.3)	<0.01
Public	4.6	42.0	(37.1–47.0)	1.3	34.3	(30.3–38.6)	<0.01
Private	13.2	46.3	(42.5–50.1)	1.5	36.1	(32.4–40.0)	<0.01
No	0.8	~	~	0.3	31.4	(23.4–40.5)	~
Usual source for care							
Yes	18.2	45.3	(41.8–48.7)	3.0	35.1	(32.3–38.1)	<0.01
No	0.4	~	~	0.1	~	~	~
Number of doctor visits in past year							
0 visits	0.2	~	~	0.0	~	~	~
1 visit	1.0	33.0	(25.3–41.7)	0.2	22.8	(15.7–32.0)	0.15
2–3 visits	6.4	46.7	(40.9–52.6)	1.0	36.3	(30.2–42.9)	<0.01
4+ visits	11.0	45.6	(41.7–49.4)	1.8	36.5	(32.7–40.4)	<0.01
Education level							
<High school	1.8	39.6	(33.0–46.6)	0.6	32.4	(25.4–40.2)	0.15
High school	4.8	45.5	(38.6–52.5)	0.6	36.6	(31.5–42.1)	0.12
Some college	7.2	48.1	(42.8–53.4)	0.9	32.3	(28.1–36.9)	<0.01
College graduate	4.8	42.6	(36.7–48.6)	0.9	38.7	(33.6–44.0)	0.01
Poverty index ratio							
<1.0	1.5	47.7	(38.7–56.9)	0.7	38.6	(31.9–45.7)	0.13
1.0–3.0	5.6	39.0	(35.0–43.1)	1.0	29.9	(24.7–35.7)	<0.01
>3.0	10.8	50.7	(45.7–55.8)	1.0	38.2	(33.3–43.5)	<0.01
Missing	0.7	27.3	(18.6–38.1)	0.4	35.3	(27.7–43.8)	0.55
Employment status							
Yes	7.4	47.0	(41.0–53.2)	1.3	34.9	(30.5–39.5)	<0.01
No	11.3	43.7	(39.5–48.1)	1.7	34.9	(30.9–39.0)	<0.01

Notes:

(1) Includes nonpregnant adults age ≥18 years with complete data on hypertension status currently taking antihypertensive medication.

(2) *N*: annual population in millions, calculated from the American Community Survey data released by NCHS, averaged across the 3 cycles. For additional information, see https://wwwn.cdc.gov/nchs/nhanes/ResponseRates.aspx#population-totals; 95% CI = 95% confidence interval around estimate.

(3) Poverty–income ratio (PIR) is based on comparison of family income with the poverty threshold determined by the US Bureau of Census. The PIR values were stratified into categories: PIR < 100% (low income), 100% ≤ PIR ≤ 300% (middle income), and ≥300% (high income) and those with missing, refused, or unknown were maintained as a category (missing).

(4) Employment status value of “Yes” refers to Employed for wages and value of “No” refers to all others.

(5) ~ Statistically unstable estimates suppressed according to National Center for Health Statistics (NCHS) Data Presentation Standards for Proportions.

(6) Reported *P* value reflects comparison of Black vs. White adult based on separate multivariate logistic regression models with blood pressure control as the dependent variable and race and the selected characteristic as the independent variables with adjustment for age and sex.

(7) White and Black adults are limited to those not selecting Hispanic as an ethnicity.

**Table 3.** Prevalence of antihypertensive medication class use and blood pressure control (<130/80 mm Hg) among White and Black adults with hypertension (<130/80 mm Hg) by medication class and number of classes used—National Health and Nutrition Examination Survey (NHANES) 2013–2018

	Prevalence of antihypertensive medication class use					Prevalence of hypertension control				
	White		Black			White		Black		
	%	(95% CI)	%	(95% CI)	*P* value	%	(95% CI)	%	(95% CI)	*P* value
Any										
Total*	55.7	(53.0–58.3)	56.4	(53.6–59.2)	<0.01	45.0	(41.7–48.3)	34.9	(32.1–37.8)	<0.01
ACEI or ARB	41.4	(39.1–43.7)	36.8	(34.0–39.7)	0.95	46.2	(42.6–49.8)	36.5	(33.5–39.6)	<0.01
BB	21.1	(19.3–23.0)	19.0	(17.2–20.9)	0.17	46.6	(40.7–52.6)	35.7	(31.0–40.6)	<0.01
CCB	14.7	(13.3–16.3)	24.2	(22.3–26.2)	<0.01	40.1	(35.2–45.2)	30.2	(26.4–34.3)	<0.01
Diuretic	23.5	(21.5–25.7)	28.5	(26.2–30.9)	<0.01	47.3	(42.9–51.8)	36.1	(32.6–39.7)	<0.01
One medication class										
Total*	21.6	(19.4–23.9)	19.1	(17.1–21.1)	0.16	41.9	(37.4–46.5)	35.1	(29.6–41.0)	0.03
ACEI or ARB	13.3	(11.6–15.2)	8.4	(7.1–9.9)	<0.01	45.0	(39.6–50.4)	36.9	(29.1–45.4)	0.07
BB	4.1	(3.2–5.2)	2.4	(1.7–3.4)	0.03	41.5	(31.6–52.2)	48.8	(30.5–67.3)	0.94
CCB	1.9	(1.4–2.6)	4.8	(3.8–6.1)	<0.01	33.4	(18.0–53.3)	30.5	(21.7–40.9)	0.43
Diuretic	2.1	(1.7–2.7)	3.0	(2.3–3.9)	0.07	33.4	(21.6–47.6)	29.0	(17.6–43.9)	0.89
Two medication classes										
Total*	20.4	(18.7–22.2)	21.1	(19.0–23.3)	0.03	46.1	(40.9–51.3)	35.2	(30.5–40.4)	<0.01
ACEI or ARB + BB	3.7	(2.9–4.8)	2.4	(1.7–3.4)	0.19	43.3	(33.6–53.5)	37.6	(24.7–52.4)	0.37
ACEI or ARB + CCB	3.2	(2.5–4.2)	3.5	(2.7–4.4)	0.26	44.7	(32.1–58.1)	31.1	(21.8–42.1)	0.07
ACEI or ARB + Diuretic	8.2	(6.8–9.9)	7.7	(6.7–9.0)	0.67	47.6	(40.0–55.3)	42.1	(34.6–49.9)	0.07
BB + CCB	0.9	(0.6–1.5)	2.0	(1.4–2.8)	0.01	51.9	(31.5–71.8)	33.2	(21.3–47.7)	0.07
BB + Diuretic	1.6	(1.2–2.1)	1.6	(1.1–2.5)	0.28	50.3	(36.1–64.4)	26.9	(13.3–47.0)	0.01
CCB + Diuretic	0.8	(0.5–1.3)	2.2	(1.7–2.8)	<0.01	23.5	(11.3—42.4)	23.8	(13.9–37.5)	1.00
Three medication classes										
Total*	9.4	(8.1–10.8)	10.5	(9.1–12.0)	0.01	47.0	(39.1–55.1)	33.6	(27.2–40.6)	0.00
ACEI or ARB + BB + CCB	1.8	(1.3–2.5)	1.1	(0.8–1.7)	0.32	28.3	(16.0–45.0)	~	~	0.53
ACEI or ARB + BB + Diuretic	3.0	(2.3–3.8)	2.5	(2.0–3.1)	0.98	53.0	(41.4–64.3)	42.7	(30.5–55.9)	0.16
ACEI or ARB + CCB + Diuretic	1.7	(1.1–2.4)	3.9	(3.1–5.0)	<0.01	~	~	36.1	(26.0–47.6)	~
BB + CCB + Diuretic	0.4	(0.2–0.7)	0.6	(0.3–0.9)	0.19	~	~	~	~	~
Four or more medication classes										
Total*	4.3	(3.3–5.6)	5.7	(4.8–6.8)	<0.01	50.9	(41.3–60.4)	35.0	(26.9–44.1)	<0.01
ACEI or ARB + BB + CCB + Diuretic	1.7	(1.2–2.4)	2.0	(1.5–2.7)	0.07	~	~	~	~	~
ACEI or ARB + BB + CCB + Diuretic + Other	0.3	(0.2–0.7)	0.9	(0.5–1.5)	<0.01	~	~	~	~	~

Abbreviations: ACEI, angiotensin-converting enzyme inhibitor; ARB, angiotensin II receptor blocker; BB, beta-blocker; CCB, calcium channel blocker.

Notes:

(1) Includes nonpregnant adults age ≥18 years with complete data on hypertension status and currently taking antihypertensive medication.

(2) Medication use is mutually exclusive, except “any use.”

(3) * Total include “other” medication class, in addition to 4 listed ones.

(4) ~ Statistically unstable estimates suppressed according to National Center for Health Statistics (NCHS) Data Presentation Standards for Proportions.

(5) Reported *P* value reflects comparison of Black vs. White adult based on separate multivariate logistic regression models with antihypertensive medication use or blood pressure control as the dependent variable and race and the selected characteristic as the independent variables with adjustment for age and sex.

(6) White and Black adults are limited to those not selecting Hispanic as an ethnicity.
