# Supplementary material for: Blood Pressure Control Among Non-Hispanic Black Adults Is Lower Than Non-Hispanic White Adults Despite Similar Treatment With Antihypertensive Medication: NHANES 2013–2018
Source: Am J Hypertens. Author manuscript; Available in PMC 2022 Jun 25. (PMC9233145; doi:10.1093/ajh/hpac011)
Supplement: SupplementaryOnlineTables [file NIHMS1810148-supplement-SupplementaryOnlineTables.docx]

**Supplemental Table 1. Prevalence of Blood Pressure Control (<140/90 mm Hg) among White and Black adults with Hypertension Currently Taking Medication—National Health and Nutrition Examination Survey (NHANES) 2013-2018**

|  | | White  (N=41.4 Million) | | | | | | | Black  (N=8.8 Million) | | | | | |  | | |  |
| --- | --- | --- | --- | --- | --- | --- | --- | --- | --- | --- | --- | --- | --- | --- | --- | --- | --- | --- |
|  | | N | | % | | | (95%CI) | N | | | | % | | (95%CI) | | P-value | |  |
| Total | | 28.9 | | 69.7 | | | (66.8-72.4) | 5.2 | | | | 58.6 | | (55.2-61.9) | | <0.01 | |  |
| Age Group (years) | |  | |  | | |  |  | | | |  | |  | |  | |  |
| 18-64 | | 16.1 | | 77.7 | | | (73.7-81.3) | 3.7 | | | | 62.0 | | (57.2-66.6) | | <0.01 | |  |
| 18-44 | | 2.7 | | 79.9 | | | (71.6-86.2) | 0.8 | | | | 62.0 | | (51.8-71.3) | | <0.01 | |  |
| 45-64 | | 13.4 | | 77.3 | | | (72.6-81.4) | 2.9 | | | | 62.0 | | (57.0-66.7) | | <0.01 | |  |
| 65+ | | 12.8 | | 61.7 | | | (57.2-65.9) | 1.5 | | | | 51.5 | | (46.5-56.5) | | <0.01 | |  |
| Sex | |  | |  | | |  |  | | | |  | |  | |  | |  |
| Men | | 14.0 | | 71.4 | | | (67.1-75.3) | | | | 2.0 | 58.2 | | (53.6-62.6) | | | | <0.01 |
| Women | | 14.9 | | 68.2 | | | (64.7-71.4) | | | | 3.1 | 58.9 | | (54.9-62.7) | | | | <0.01 |
| Health Insurance | |  | |  | | |  |  | | | |  | |  | |  | |  |
| Yes | | 27.6 | | 69.7 | | | (66.7-72.5) | 4.8 | | | | 59.7 | | (56.5-62.8) | | <0.01 | |  |
| Public | | 7.3 | | 66.4 | | | (61.0-71.4) | 2.2 | | | | 56.0 | | (51.0-60.8) | | <0.01 | |  |
| Private | | 20.3 | | 70.9 | | | (67.2-74.4) | 2.6 | | | | 63.2 | | (58.5-67.7) | | <0.01 | |  |
| No | | 1.2 | | ~ | | | ~ | 0.4 | | | | 48.4 | | (36.4-60.5) | | ~ | |  |
| Usual source for care | |  | |  | | |  |  | | | |  | |  | |  | |  |
| Yes | | 28.0 | | 69.6 | | | (66.8-72.3) | 5.0 | | | | 58.6 | | (55.3-61.9) | | <0.01 | |  |
| No | | 0.8 | | ~ | | | ~ | 0.2 | | | | ~ | | ~ | | ~ | |  |
| Number of doctor visits in past year | | | | |  | |  |  | | | |  | |  | |  | |  |
| 0 visits | | 0.3 | | ~ | | | ~ | 0.0 | | | | ~ | | ~ | | ~ | |  |
| 1 visit | | 1.9 | | 59.5 | | | (48.0-70.0) | 0.5 | | | | 51.8 | | (42.2-61.3) | | 0.29 | |  |
| 2-3 visits | | 9.9 | | 72.1 | | | (67.7-76.0) | 1.6 | | | | 60.0 | | (53.9-65.7) | | <0.01 | |  |
| 4+ visits | | 16.8 | | 69.8 | | | (65.9-73.5) | 3.0 | | | | 60.0 | | (55.3-64.5) | | <0.01 | |  |
| Education level | |  | |  | | |  |  | | | |  | |  | |  | |  |
| <High School | | 3.1 | | 71.0 | | | (63.1-77.8) | 1.0 | | | | 53.3 | | (45.3-61.2) | | <0.01 | |  |
| High School | | 7.7 | | 67.9 | | | (62.0-73.2) | 1.4 | | | | 58.2 | | (52.5-63.7) | | <0.01 | |  |
| Some College | | 10.2 | | 68.2 | | | (63.5-72.5) | 1.7 | | | | 59.1 | | (54.4-63.7) | | <0.01 | |  |
| College Graduate | | 7.8 | | 73.3 | | | (68.1-77.8) | 1.1 | | | | 63.7 | | (57.0-70.0) | | <0.01 | |  |
| Poverty index ratio | |  | |  | | |  |  | | | |  | |  | |  | |  |
| <1.0 | | 2.2 | | 70.8 | | | (58.8-80.5) | 1.1 | | | | 56.4 | | (48.9-63.5) | | 0.03 | |  |
| 1.0-3.0 | | 8.9 | | 62.3 | | | (58.4-66.0) | 1.8 | | | | 53.5 | | (47.3-59.6) | | <0.01 | |  |
| >3.0 | | 16.2 | | 75.7 | | | (71.8-79.1) | 1.7 | | | | 66.1 | | (60.6-71.2) | | <0.01 | |  |
| Missing | | 1.6 | | 60.2 | | | (49.5-70.0) | 0.6 | | | | 59.9 | | (49.1-69.8) | | 0.33 | |  |
| Employment status | |  | |  | | |  |  | | | |  | |  | |  | |  |
| Yes | | 11.8 | | 75.2 | | | (69.9-79.8) | 2.3 | | | | 60.2 | | (55.6-64.6) | | <0.01 | |  |
| No | | 17.1 | | 66.4 | | | (62.8-69.8) | 2.9 | | | | 57.4 | | (53.1-61.6) | | <0.01 | |  |

Notes:

1) Includes non-pregnant adults age **≥**18 years with complete data on hypertension status and currently taking anti-hypertensive medication.

2) N: Annual population in millions, calculated from the American Community Survey data released by NCHS, averaged across the 3 cycles. For additional information, see: <https://wwwn.cdc.gov/nchs/nhanes/ResponseRates.aspx#population-totals>.

3) Poverty-income ratio is based on comparison of family income with the poverty threshold determined by the US Bureau of Census. The PIR values were stratified into four categories: PIR < 100% (low income), 100% ≤ PIR ≤ 300% (middle income), and ≥300% (high income) and those with missing, refused, or unknown PIR were maintained as a category (missing).

4) Employment Status value of ‘Yes’ refers to Employed for wages and value of ‘No’ refers to all others.

5) ~ Statistically unstable estimates suppressed according to NCHS Data Presentation Standards for Proportions.

6) Reported P-value reflects comparison of Black vs. White adult based on separate multivariate logistic regression models with blood pressure control as the dependent variable and race and the selected characteristic as the independent variables with adjustment for age and sex.

7) White and Black adults is limited to those not selecting Hispanic as an ethnicity.

**Supplemental Table 2. Prevalence of Anti-Hypertensive Medication Class Use and Blood Pressure Control (<140/90 mm Hg) among White and Black adults with Hypertension (<140/90 mm Hg) by Medication Class and Number of Classes Used -- National Health and Nutrition Examination Survey (NHANES) 2013-2018**

|  | Total | | | | | Total | | | | |
| --- | --- | --- | --- | --- | --- | --- | --- | --- | --- | --- |
|  | NH White | | NH Black | |  | NH White | | NH Black | |  |
|  | % | (95%CI) | % | (95%CI) | P_value | % | (95%CI) | % | (95%CI) | P_value |
| Any |  |  |  |  |  |  |  |  |  |  |
| Total* | 76.4 | (73.6-79.0) | 75.1 | (72.5-77.5) | 0.39 | 69.7 | (66.8-72.4) | 58.6 | (55.2-61.9) | <0.01 |
| ACEI or ARB | 56.9 | (54.2-59.5) | 49.0 | (46.1-52.0) | 0.01 | 71.8 | (68.6-74.9) | 58.4 | (54.7-62.0) | <0.01 |
| BB | 29.0 | (26.5-31.6) | 25.3 | (22.9-27.8) | 0.92 | 68.2 | (63.2-72.8) | 58.3 | (52.8-63.6) | <0.01 |
| CCB | 20.2 | (18.2-22.5) | 32.2 | (29.9-34.6) | 0.00 | 62.6 | (57.1-67.9) | 57.8 | (52.6-62.9) | <0.01 |
| Diuretic | 32.3 | (29.5-35.3) | 38.0 | (35.1-41.0) | 0.00 | 71.2 | (66.8-75.2) | 58.9 | (55.0-62.7) | <0.01 |
| One Medication class |  |  |  |  |  |  |  |  |  |  |
| Total* | 29.6 | (27.0-32.4) | 25.4 | (22.9-28.0) | 0.00 | 68.9 | (63.8-73.5) | 61.4 | (54.6-67.7) | 0.03 |
| ACEI or ARB | 18.3 | (16.1-20.7) | 11.1 | (9.5-13.0) | 0.00 | 74.7 | (68.6-79.9) | 61.5 | (53.3-69.0) | 0.07 |
| BB | 5.6 | (4.5-7.1) | 3.2 | (2.2-4.5) | 0.01 | 62.0 | (50.9-72.0) | ~ | ~ | ~ |
| CCB | 2.6 | (1.9-3.5) | 6.4 | (5.1-8.1) | 0.00 | ~ | ~ | 63.7 | (53.4-72.9) | ~ |
| Diuretic | 2.9 | (2.3-3.7) | 4.0 | (3.0-5.2) | 0.24 | ~ | ~ | ~ | ~ | ~ |
| Two Medication class |  |  |  |  |  |  |  |  |  |  |
| Total* | 28.0 | (25.6-30.5) | 28.1 | (25.6-30.7) | 0.61 | 71.6 | (68.4-74.7) | 57.6 | (52.1-62.9) | <0.01 |
| ACEI or ARB + BB | 5.1 | (4.0-6.5) | 3.2 | (2.3-4.5) | 0.08 | 69.6 | (60.2-77.6) | ~ | ~ | 0.37 |
| ACEI or ARB + CCB | 4.5 | (3.4-5.8) | 4.6 | (3.7-5.8) | 0.53 | 72.7 | (58.7-83.3) | 57.7 | (44.7-69.7) | 0.07 |
| ACEI or ARB + Diuretic | 11.3 | (9.3-13.6) | 10.3 | (9.0-11.8) | 0.17 | 75.5 | (69.4-80.7) | 59.2 | (50.3-67.5) | 0.07 |
| BB +CCB | 1.3 | (0.8-2.0) | 2.7 | (1.8-3.8) | 0.02 | ~ | ~ | ~ | ~ | ~ |
| BB + Diuretic | 2.2 | (1.6-2.9) | 2.2 | (1.4-3.3) | 0.52 | ~ | ~ | ~ | ~ | ~ |
| CCB + Diuretic | 1.1 | (0.7-1.8) | 3.0 | (2.3-3.8) | 0.00 | ~ | ~ | ~ | ~ | ~ |
| Three Medication class |  |  |  |  |  |  |  |  |  |  |
| Total* | 12.9 | (11.2-14.7) | 14.0 | (12.2-16.0) | 0.04 | 65.0 | (56.6-72.5) | 56.1 | (49.9-62.2) | 0.00 |
| ACEI or ARB + BB + CCB | 2.5 | (1.8-3.4) | 1.5 | (1.0-2.3) | 0.20 | ~ | ~ | ~ | ~ | 0.53 |
| ACEI or ARB + BB + Diuretic | 4.1 | (3.1-5.3) | 3.3 | (2.7-4.2) | 0.66 | 67.5 | (56.5-76.8) | ~ | ~ | 0.16 |
| ACEI or ARB + CCB + Diuretic | 2.3 | (1.6-3.3) | 5.2 | (4.1-6.7) | 0.00 | ~ | ~ | 61.2 | (50.7-70.8) | ~ |
| BB + CCB + Diuretic | 0.5 | (0.3-0.9) | 0.8 | (0.5-1.2) | 0.27 | ~ | ~ | ~ | ~ | ~ |
| Four or more Medication classes |  |  |  |  |  |  |  |  |  |  |
| Total* | 5.9 | (4.6-7.7) | 7.7 | (6.5-9.1) | 0.00 | 74.9 | (67.2-81.4) | 57.7 | (47.6-67.1) | <0.01 |
| ACEI or ARB + BB + CCB + Diuretic | 2.3 | (1.7-3.3) | 2.6 | (2.0-3.6) | 0.14 | ~ | ~ | ~ | ~ | ~ |
| ACEI or ARB + BB + CCB + Diuretic + Other | 0.4 | (0.2-0.9) | 1.2 | (0.7-2.0) | 0.01 | ~ | ~ | ~ | ~ | ~ |

Notes:

1) Includes non-pregnant adults age >=18 years with complete data on HTN status and prescription medication use.

2) Reported P-value reflects comparison of Black vs. White adult based on separate multivariate logistic regression models with antihypertensive medication use or blood pressure control as the dependent variable and race and the selected characteristic as the independent variables with adjustment for age and sex.

3) Medication use is mutually exclusive, except 'any use'.

4) *: Total include 'other' medication class, in addition to 4 listed ones.

| 5) ~: Suppressed according to NCHS Data Presentation Standards for Proportions. |  |
| --- | --- |
